# Mood As Cumulative Expectation Mismatch: A Test of Theory Based on Data from Non-verbal Cognitive Bias Tests

**DOI:** 10.3389/fpsyg.2017.02197

**Published:** 2017-12-14

**Authors:** Camille M. C. Raoult, Julia Moser, Lorenz Gygax

**Affiliations:** ^1^Centre for Proper Housing of Ruminants and Pigs, Federal Food Safety and Veterinary Office FSVO, Agroscope, Ettenhausen, Switzerland; ^2^Animal Welfare Division, Veterinary Public Health Institute, Vetsuisse Faculty, University of Bern, Bern, Switzerland

**Keywords:** affective states, mood, cognitive bias, judgment bias, animal welfare

## Abstract

Affective states are known to influence behavior and cognitive processes. To assess mood (moderately long-term affective states), the cognitive judgment bias test was developed and has been widely used in various animal species. However, little is known about how mood changes, how mood can be experimentally manipulated, and how mood then feeds back into cognitive judgment. A recent theory argues that mood reflects the cumulative impact of differences between obtained outcomes and expectations. Here expectations refer to an established context. Situations in which an established context fails to match an outcome are then perceived as mismatches of expectation and outcome. We take advantage of the large number of studies published on non-verbal cognitive bias tests in recent years (95 studies with a total of 162 independent tests) to test whether cumulative mismatch could indeed have led to the observed mood changes. Based on a criteria list, we assessed whether mismatch had occurred with the experimental procedure used to induce mood (mood induction mismatch), or in the context of the non-verbal cognitive bias procedure (testing mismatch). For the mood induction mismatch, we scored the mismatch between the subjects’ potential expectations and the manipulations conducted for inducing mood whereas, for the testing mismatch, we scored mismatches that may have occurred during the actual testing. We then investigated whether these two types of mismatch can predict the actual outcome of the cognitive bias study. The present evaluation shows that mood induction mismatch cannot well predict the success of a cognitive bias test. On the other hand, testing mismatch can modulate or even inverse the expected outcome. We think, cognitive bias studies should more specifically aim at creating expectation mismatch while inducing mood states to test the cumulative mismatch theory more properly. Furthermore, testing mismatch should be avoided as much as possible because it can reverse the affective state of animals as measured in a cognitive judgment bias paradigm.

## Introduction

In the last years, interest in affective states in animals has increased (Boissy et al., 2007; Veissier and Boissy, 2007) because these states are thought to reflect welfare from the perspective of the animals (Kendrick, 2007; Yeates and Main, 2008). Also, affective states may be highly relevant for the proximate control of behavior (Gygax, 2017). Here, we use “affective states” as a general term encompassing any affective experience. It is assumed that different types of affective states are relevant for different periods of time (Spruijt et al., 2001; Posner et al., 2005; Mendl et al., 2009). Nevertheless, all states can be characterized by their valence dimension (positive vs. negative; Kron et al., 2015). Emotions, that is, short-term affective states, refer to adaptive processes as part of the basic mechanism that allows animals to avoid punishment and reach reward when directly confronted with internal and external stimuli (Panksepp, 1994; Rolls, 2000). According to Schachter and Singer (1962), subjective emotional response to a situation is thought to occur through a process of appraisal after a series of stimulus checks (e.g., suddenness, familiarity, predictability, pleasantness). Emotions are relatively intense and short-lived affective reactions. Mood differs from emotions in that it represents a state that typically lasts longer over a moderate period of time such as days or weeks (Eldar et al., 2016). Mood is less tightly linked to particular events and is thought to reflect the cumulative impact of multiple stimuli (e.g., depression; Mendl et al., 2010b; Nettle and Bateson, 2012; Eldar et al., 2016). In some of the literature, the terms affective *states* and *traits* are used. Affective *states* are characterized by situational or contextual factors observable as within-individual variability. Affective *traits* on the other hand are constant over long periods of time and specific to each individual (Luthans and Youssef, 2007; Kluember et al., 2009; Faustino et al., 2015). Traits encompass, e.g., how individuals deal with affective situations in general and can be considered part of their personality. We refer to mood as an affective *state* that persists over some time rather than being a fleeting response to a single event (Luthans and Youssef, 2007; Kivumbi, 2011; Faustino et al., 2015).

Mood is likely to be relevant to understand animal and human behavior because it seems that subjects in a more positive mood state deal more easily with negative short-term experiences (i.e., they are more “optimistic”, Mendl et al., 2009, 2010b). A positive mood might therefore alleviate single negative events and stabilize emotional reactions, that is, subjects will react also less strongly to positive events (Mendl et al., 2009, 2010b; Muehlemann et al., 2011; Laeger et al., 2012; Reefmann et al., 2012). On the contrary, a negative mood state might taint experiences quite generally (Rottenberg et al., 2005; Grippo and Johnson, 2009, Groenwold et al., 2013). Mood states become particularly important and visible in situations of ambiguity and uncertainty where the expectation of a subject (optimism and pessimism) is pre-shaped to a smaller extent by the circumstances of the situation (Schachter and Singer, 1962; Mendl et al., 2009; Roelofs et al., 2016).

Human studies have shown that affective states influence cognitive processes, therefore a cognitive bias can indicate a specific mood state (Paul et al., 2005). Cognitive biases influence emotional responses by altering the processing of affective information (i.e., a shift in judgment; Mendl et al., 2009). This becomes important and visible specifically in situations of ambiguity and uncertainty because the decision to be taken is shaped less clearly by the situation itself (Roelofs et al., 2016). It is thought that a more negative mood engenders negative judgments (Kavanagh and Bower, 1985; Headey and Veenhoven, 1989; Mayer et al., 1992). Moreover, mood affects decision-making (Brydges et al., 2012; Murphy et al., 2015), risk-taking (Halek and Eisenhauer, 2001), learning (Vögeli et al., 2014), and motivation (Rygula et al., 2015).

Recently, Eldar et al. (2016) presented the first concise and coherent theory on mood. They argued that mood reflects the cumulative impact of differences between obtained outcomes and expectations in an established context (i.e., mismatches). Often, it is impossible to determine what a non-verbal individual expects. We use the term ‘expectation’ to reflect a situation in which the stage has been set for the individual to anticipate a particular outcome that may or may not match the observed outcome, without making any assumption about the internal mental state of the individual. That is, the emotional mismatches occurring in a subject’s life are thought to influence their future mood, thanks to positive or negative feedback. In turn, mood biases the way that outcomes are perceived (i.e., valuation of subsequent outcomes, Huntsinger, 2012; Eldar et al., 2016) and this bias affects learning about those outcomes. In this sense, mood represents the “overall momentum” of recent unexpected outcomes (Eldar et al., 2016). In other terms, mood does not result from cumulative recent rewards or punishments but depends whether cumulative recent outcomes were better or worse than expected. Behavioral and neural findings suggest that mood biases the perception of reward outcomes such that outcomes are perceived better than they are when a subject is in a good mood, and worse than they really are when the subject is in a bad mood (Eldar et al., 2016). In humans, Headey and Veenhoven (1989) suggest that a positive mood state results in an appreciation of the life-as-a-whole, helps to cope with problems and can even alleviate negative events. Yet, it can also induce a rosy perspective, that is, an optimistic outlook which may also, for example, masks the individual personal problems (Headey and Veenhoven, 1989). Mathews and MacLeod (2005) and Gotlib and Joormann (2010) agree in saying that a negative mood state is characterized by pessimistic automatic thoughts and biases in attention, interpretation and memory towards negative situations and events.

To measure mood in animals, the non-verbal cognitive judgment bias test has been introduced as the standard approach (Harding et al., 2004; Mendl et al., 2009). In this test, subjects are trained to link a cue with a rewarding event and another cue (on the same physical axis) with an aversive event. Subjects are then confronted with ambiguous cues. If the behavioral reaction to ambiguous cues is more similar to that shown in response to the rewarding cue the animal is considered to be in a more optimistic state. On the contrary, if the reaction reflects the one shown in response to the negative cue, the animal is considered being more pessimistic. In many studies implementing a cognitive bias test, an attempt is made to manipulate the mood state of the animals in some form of mood induction (Bethell, 2015). For instance, mood can be manipulated in animals by multiple short-term changes (e.g., husbandry interventions, social introductions, chronic stress), moderately long-term modification (e.g., housing conditions) and therapeutics drugs (Mendl et al., 2009; Bethell, 2015; Bethell et al., 2016; Hales et al., 2016). These attempts include changes in the internal or external environment that deteriorate or improve conditions. The basis for such claims is based more on common sense rather than deep prior knowledge. The effectiveness of the mood induction was shown to be higher if different types of manipulations were combined (Westermann et al., 1996). Mendl et al. (2009) found that more diverse and unrelated manipulations (e.g., unpredictable housing conditions) resulted in predicted changes in judgment bias more often. In this way, multiple short-term manipulations can be thought of producing mismatches that would then causally influence mood. Also, the manipulations have not usually been shaped by the aim of producing mismatches that are viewed as the cause of mood changes by Eldar et al. (2016). In many studies, in addition to the “treated” group, a control group is used to allow for comparisons (see Baciadonna and McElligott, 2015). However, despite the number of publications suggesting that this approach is now well established to probe moderately to long-term mood in various species (Mendl et al., 2009), the results of experiments are still not unequivocal (Wichman et al., 2012; Anderson et al., 2013; Scollo et al., 2014; Guldimann et al., 2015; Roelofs et al., 2016). Results are not always in line with the prediction, sometimes even showing that mood had been influenced in the contrary direction as expected (e.g., see Doyle et al., 2010; Burman et al., 2011; Brydges et al., 2012; Parker et al., 2014). For example, it has been hypothesized that restraining individuals will lead to a more negative mood and, accordingly result in a more negative judgment of ambiguous cues. However, these individuals were reported to display a more positive judgment of the ambiguous cues than control individuals (Doyle et al., 2010; Briefer Freymond et al., 2014; Wheeler et al., 2015; Horváth et al., 2016). Additionally, similarly valenced states, such as depression, anxiety and fear, do not always have the same effects on a situation cognitive appraisal (Lerner and Keltner, 2000). Indeed, little is known what exactly causes changes in mood and it is not well understood how mood then influences cognitive judgment (Mendl et al., 2009). It remains elusive how short-term emotional reactions accumulate and feed back into the perception of outcomes (Eldar and Niv, 2015).

Here, we take advantage of the large number of studies published on non-verbal cognitive bias tests in recent years that include an experimental manipulation of mood, to test whether cumulative mismatch could indeed have led to the observed results in the cognitive bias test (Eldar et al., 2016). To do so, we extended the list of studies presented by Gygax (2014) and Knapp et al. (2015). In 95 studies, we reviewed the conditions used to manipulate mood in respect to whether mismatch was likely to have occurred at different times in the timeline. Based on a criteria list, we assessed whether prior to the test a “mood induction mismatch” occurred, i.e., whether the experimental procedure used to induce mood contained aspects that can be viewed as creating cumulative mismatch. In cognitive bias tests, the testing procedure itself is thought to have a potential influence on the animals’ reaction in the test (e.g., Doyle et al., 2010). Therefore, we additionally assessed whether at the time of testing a “testing mismatch” occurred, i.e., whether the testing procedure itself could have induced a mismatch based on a second list of criteria. We then investigated whether these two types of mismatch could predict the actual outcome and interpretation of the cognitive bias test in the collected studies. This outcome was scored as either fitting with the hypothesis (a cognitive bias could be supported by the data as expected by the author(s)), none (no cognitive bias could be supported), or contrary to the hypothesis (bias in the unexpected direction was supported by the data). The author(s)’ hypotheses were mainly based on human literature. They seem reasonable in our view and were presumably backed-up by the reviewers and editor of these papers at the time of publication. We focused on quantitative tests of these hypotheses made at the level of a group of subjects (either comparing a ‘treated’ group with a control group or testing the same individuals in different conditions). According to Eldar et al. (2016), we expected that the experimental mood induction would be successful if it included cumulative mismatch (“mood induction mismatch”). This success may be modulated if the cognitive bias testing itself produced some additional mismatch (“testing mismatch”).

## Materials and Methods

We used the list of published studies involving a cognitive bias approach by Gygax (2014) and Knapp et al. (2015), as a starting point. We complemented the list by studies published, or in press and available online, between the time point of the search in Gygax (2014) and July 31st 2017. Studies were identified, as in Gygax (2014), by searching the Web of Science^1^ using a cited-reference search for ‘Harding et al. (2004)’ combined with (OR) the key word combination ‘[(cognitive AND bias AND welfare) OR (judgment AND bias AND welfare)]’. We used an additional search consisting of a cited-reference search for ‘Harding et al. (2004)’ combined with (AND) the key word combination ‘[(cognitive AND bias) OR (judgment AND bias)]’, a third search with the key word combination ‘(affective AND state) AND [(cognitive AND bias) OR (judgment AND bias)]’, and a fourth search with the key word combination ‘(state AND affect) AND [(cognitive AND bias) OR (judgment AND bias)]’. We also conducted the latter search using ‘(affective AND state) OR ((cognitive AND bias) OR (judgment AND bias))’. With this search, >20’000 hits were reached. The first dozens of hits were irrelevant to a large extent and therefore, we did not further pursue this line of search. For this review, only studies that attempted to actively induce mood or in which mood was independently inferred (e.g., based on self-reports, behavioral, or physiological data) were considered. Other methodological studies were excluded from this analysis, as well as publications including fewer than 4 subjects (per treatment group), because they are lacking the necessary degrees of freedom for a quantitative statistical evaluation at group level. Up to the day of the final literature search, we found a total of 95 studies including 162 independent cognitive bias tests that reflected our sample size (for a list of the studies see Supplementary Material).

### Outcome of the Cognitive Bias Test

The outcome of the studies was assessed and categorized as ‘fitting’ with the hypothesis when there was evidence for the hypothesis that was originally formulated by the authors in the source papers (and presumably backed-up by the reviewers and editor of these papers). These hypotheses were also coherent with what one would assume based on the literature. In these cases, a cognitive bias based on the mood induction was supported by the data. The outcome was deemed ‘contrary’ when there was evidence in the contrary direction to the original hypothesis, that is, the cognitive bias was inverse to the one expected, or as ‘none’ if no evidence for a difference in cognitive bias was found. Here, we took a *p*-value of *p* ≤ 0.05 (statistically “significant”) at any of the ambiguous cue values in the original study as the criterion for fitting or contrary outcomes.

### Mood Induction Mismatch

The cumulative mismatch according to the mood induction method (‘mood induction mismatch’) was assessed and categorized either as a negative mismatch, no mismatch, or positive mismatch. Indeed, an attempt to manipulate mood in our sample of studies does not necessarily lead to a mood induction mismatch, because subjects need to experience differences between obtained outcomes and expectations to qualify for a mismatch. Here, we base our judgment of an expectation solely on the fact that the subjects experienced certain aspects of their environment that were experimentally changed for mood induction and accordingly caused a potential mismatch. In order to be considered cumulative, the experimental manipulations needed to be either repeated at least twice or applied during several days, excepted for pharmacological treatments which mimic the effect of a cumulative mismatch. Pharmacological treatments are used to more directly manipulate the animal’s ‘internal (neural) state’ (Mendl et al., 2009). This approach is also used to validate the physiological mechanism by showing that the pharmacological manipulation leads to the predicted judgment bias (e.g., Peet and Peters, 1995; Doyle et al., 2011a; Destrez et al., 2012). On the other hand, the use of an *a priori* appropriate pharmacological treatment can reverse the effect of an environmental manipulation on bias (e.g., Karagiannis et al., 2015). Also, behavioral and physiological measures can be used to provide insight into the past subjective experiences of individuals (Verbeek and Lee, 2014).

Additionally, we wanted originally to take into account the mismatch intensity (e.g., number of repetitions, treatment intensity and the duration of the treatment phase). However, the exact reporting of these measures was often lacking and could therefore not be consistently quantified across studies. Because these quantitative aspects were highly diverse between the studies, we did not make an attempt to integrate them in, for example, several levels of scoring negative and positive mismatch. The simple scoring system used here, seemed to reflect the studies most consistently.

A negative mood induction mismatch was scored when experimental conditions were likely to induce a clear difference between what subjects expected and what actually happened. To be considered negative, subjects’ expectations should be better than the obtained outcomes due to mood manipulations, i.e., the manipulation for mood induction was worse than expected by the tested subjects. In these cases, the subjects’ previous conditions were often deteriorated. Such conditions included at least one and potentially several of the following points (with exemplary studies):

• a change in environment such as a reduction in the level of enrichment (perches removed, Bateson and Matheson, 2007), reducing space allowance (animals housed at high density, Douglas et al., 2012; metabolic cages, Barker et al., 2016), introducing unpredictable housing conditions (unattainable food, sudden unfamiliar noise, Doyle et al., 2011b; unpredictable aversive events occurring at different times of the day, Destrez et al., 2013) or deteriorating lighting conditions (reverse light/dark cycle, Harding et al., 2004),
• a decrease in the quality of human-animal interactions such as rough human contact (talking loudly and abruptly, turning animals on their back, immobilization, catching and picking an animal up, hitting the back of an animal with the hand, shaking aluminum leaves near it, Brajon et al., 2015), reduction in positive human contact (absence of the owner, Muller et al., 2012; no human interaction, Brajon et al., 2015), or unpleasant handling (tattoing, foot bath treatment, transportation, Doyle et al., 2011b; shearing, Sanger et al., 2011; tilting cage, Chaby et al., 2013; dehorning, Daros et al., 2014),
• inter-specific contact with a potential predator in a situation that subjects had previously experienced as safe (animals vigorously shaken simulating a predator attack, Bateson et al., 2011; animal confronted with predator, Doyle et al., 2011b),
• a decrease or change in intra-specific contact after subjects had experienced a stable social situation, such as isolation (Salmeto et al., 2011), separation (from the dam, Daros et al., 2014), mixing with unfamiliar congeners (introducing a stranger of the same species, Harding et al., 2004; daily social defeat in an resident–intruder paradigm, Papciak et al., 2013), or unpleasant odor (conspecifics blood/urine odors from slaughterhouse, Destrez et al., 2013),
• a pharmacological treatment likely to mimic one of the conditions mentioned above such as a stress-inducing drug (combined noradrenergic-glucocorticoid injection, Enkel et al., 2010; administration of p-Chlorophenylalanine, Doyle et al., 2011a),
• the recording of a specific behavior that is likely to reflect previous suffering or anxiety such as stereotypy (head twirls, Pomerantz et al., 2012; back-flipping, Novak et al., 2016), fearfulness (animal afraid by a novel object, Carreras et al., 2016), rumination (anxious person, Schick et al., 2013) or separation distress (in dogs, Mendl et al., 2010a) that were thought to be the effect of conditions as mentioned above,
• a history of poor welfare thought to have consisted of a combination of conditions as listed above (kennel housed animals at rehoming center, Titulaer et al., 2013),
• a clinically depressed state such as a genetic animal model of depression (congenitally helpless animals, Richter et al., 2012; high initial anxiety phenotype, Kloke et al., 2014) or depression in humans (Schick et al., 2013) that otherwise develop if subjects are confronted with conditions as listed above.

A positive mood induction mismatch was scored when experimental conditions for mood manipulation were likely to surpass the subjects’ expectations. To be considered positive, subjects’ expectations should be worse than the obtained outcomes due to mood manipulation, i.e., the manipulation for mood induction resulted in outcomes better than expected by the tested subjects. In these cases, the subjects’ previous conditions were often improved. Such conditions included at least one and potentially several of the following points (with exemplary studies):

• a change in environment such as providing enrichment (shredded paper nesting material, Burman et al., 2008; perches, Matheson et al., 2008; attractive items in respect to playing behavior, Keen et al., 2014), increasing space allowance (animals housed in a multilevel caging system, Wheeler et al., 2015), introducing predictable housing conditions (light signal before food, Destrez et al., 2014), or increasing thermal comfort (animal under high temperature, Deakin et al., 2016),
• improve human–animal interactions such as gentle human handling after they had experienced neutral or negative interactions only (tickling the animals, Rygula et al., 2012; brushing the animals, Destrez et al., 2014; habituating the animals to the human presence, talking softly to them, Brajon et al., 2015),
• improve intra-specific contact such as pasturing with conspecifics (access to pastures and conspecifics after being kept singly, Löckener et al., 2016),
• introduce rewarding events such as offering food items or increasing cognitive demand (learning to search food in a maze, Burman et al., 2011),
• a pharmacological treatment likely to mimic one of the conditions mentioned above such as an antidepressant (Citalopram, Rygula et al., 2014), anxiolytic (Diazepam, Destrez et al., 2012), or stimulant drug (d-Amphetamine, Rygula et al., 2014).

Sixteen studies used negative and positive mismatch as defined above in the same study and applied the two types of mismatches to two groups that were then compared. These studies were too rare to consider the impact of negative and positive mismatch independently within a given study. If we had considered these few cases with two different mismatches, the predictor variable mood induction mismatch would have become a within-study variable and no longer a between-study variable. The latter was more appropriate for the large majority of the studies, though. Therefore, studies including a positive manipulation in one treatment group along with a negative manipulation in another treatment group were scored as negative mismatch only, because negative events are often thought to be more ecologically relevant.

When none of the previous criteria was observed, no mood induction mismatch was scored (18 independent tests in total).

### Testing Mismatch

The mismatch according to the testing procedure for the cognitive bias itself (‘testing mismatch’) was assessed and categorized also as either a negative mismatch, no mismatch, or a positive mismatch. Criteria for testing mismatches were based on studies that emphasized such a potential effect (e.g., Burman et al., 2009; Brilot et al., 2010; Doyle et al., 2010). We then applied these criteria also to all other publications in our sample. This mismatch was related to the treated group only (not the control group).

A negative testing mismatch was considered when one or several of the following events took place in the cognitive bias test (with exemplary studies):

• a learning effect, that is, a habituation about the outcomes of ambiguous cues in term of decreasing responses to unrewarded probes because they had the same effect as the negative probes (decrease the frequency, Doyle et al., 2011b; increase the latency, Murphy et al., 2013),
• a negative anticipation of the test because the treated subjects were less motivated to perform the test than the control ones. For instance, in Burman et al. (2011), the treated subjects had a food reward before the test and were then less motivated than the control animals to search food in the maze,
• when one of the listed points for the negative mood induction mismatch was applied during the test.

A positive testing mismatch was considered when at least one of the following events appeared (with exemplary studies):

• a positive anticipation of the test (motivation related to anticipation of reward, Briefer Freymond et al., 2014),
• when the judment bias test was less stressful than the mood induction procedure (release from a negative state experienced during treatment, Doyle et al., 2010; stimulated during the test as a consequence of unpredictable housing treatment, Parker et al., 2014),
• when one of the listed points for the positive mood induction mismatch was applied during the test.

When none of the previous criteria was observed, no testing mismatch was scored. As previously, when several mismatches occurred, no attempt was made to integrate them in, for example, several levels of scoring negative and positive mismatch.

### Inter-Observer Agreement

The outcome of the cognitive bias test was assessed by one scorer only (CR). In contrast, the mood induction mismatch and the testing mismatch were independently assessed by two scorers (CR, JM). The first scorer (CR) had access to the full publications and developed the list of criteria from an initial set while scoring. In contrast, the second scorer (JM) had access to the Methods section of the publications only. Moreover, she scored the studies’ mismatches solely based on the finalized criteria listed above. This scorer was additionally blinded for the studies’ background and in respect to our hypotheses. The aim was to assess the studies with the least possible bias. The assessments of the two scorers were then compared.

The scorers differed in respect to 47 independent tests (41 studies, respectively) in the assignment of the mismatches. However, the number of independent tests per study and some aspect of the testing could not be properly determined in 22 and 11 cases, respectively, based on the Methods section alone. For the final 14 cases, the second scorer (JM) had overlooked one of the listed criteria. Therefore, when information was completed and all items of the criteria list were considered the two observers fully agreed.

### Statistic

**FIGURE 1:**
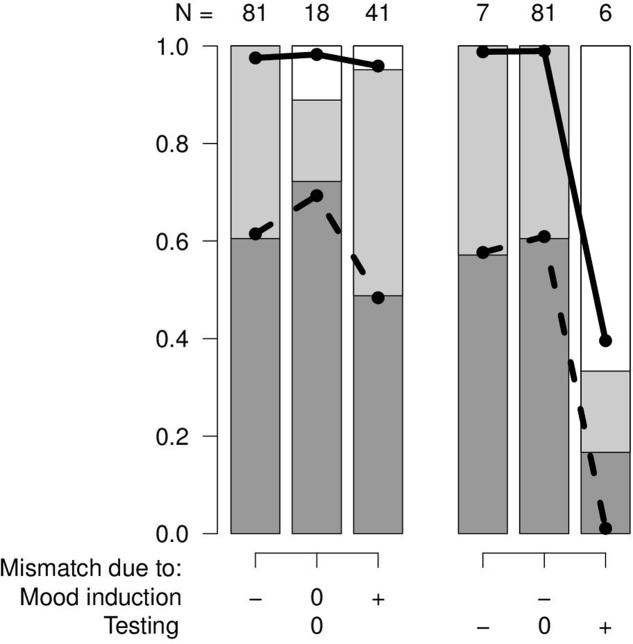
Success of independent cognitive bias tests (dark gray: fitting with the hypothesis, light gray: none, white: contrary to the hypothesis) in function of the mood induction mismatch (–: negative, 0: none, +: positive) and the testing mismatch (–: negative, 0: none, +: positive). *N*: number of independent tests in each combination. Black lines: model estimates that reflect the probability of the switches from one level in the outcome variable to the next.

Statistical analyses were performed in R version 3.3.2 (R Core Team, 2016) using a proportional odds model (package MASS, Ripley et al., 2016). The outcome variable was the success of the cognitive bias test (ordered three-level factor: contrary-none-fitting). Explanatory variables were the mood induction mismatch (three-level factor: negative-none-positive) and the testing mismatch (three-level factor: negative-none-positive). All the combinations of mood induction mismatch and testing mismatch did occur. Nevertheless, four of the combinations were observed only at very low numbers (3 or less observations for the negative and positive testing mismatch combined with either no or positive mood induction mismatch). Accordingly, a full model did not seem meaningful because the counts of the different potential outcomes in these rare combinations need to be considered random to a large extent. Therefore, the effect of the mood induction mismatches was evaluated for those tests only that had no testing mismatch (**Figure 1**, left). Also, the effects of the testing mismatch were evaluated in those studies only that had a negative mood induction mismatch (**Figure 1**, right). To support model interpretation, *p*-values were calculated for the predictors using a likelihood-ratio test.

## Results

In the studies without testing mismatch, there was a slightly higher proportion of successful cognitive bias studies when there was no cumulative mood induction mismatch compared with either a positive or a negative mismatch. Even though this difference was around 20% it was not clearly supported by a low *p*-value (χ^2^_2_ = 2.87, *p* = 0.24; **Figure 1**, left).

With a negative cumulative mood induction mismatch, the success of cognitive bias studies decreased from no testing mismatch and a negative testing mismatch to a positive testing mismatch (χ^2^_2_ = 19.04, *p* = 0.00007; **Figure 1**, right).

## Discussion

The aim of this review was to test whether cumulative mismatch could indeed have led to the observed decision biases in cognitive bias studies. This would then indicate that the cumulative mismatch had led to a change in mood. We expected that the experimental mood induction would be successful if it included cumulative mismatch. In these cases, we anticipated a cognitive bias test outcome fitting with the hypothesis. Yet, mood induction mismatch did not strongly predict success of the cognitive bias test. The effect of the mood induction mismatches could be evaluated for those tests only that had no testing mismatch. This implies that, as far as we know, the cognitive bias test outcome results were not influenced by a testing mismatch therefore increasing the probability to see an effect of mood induction mismatch. If at all, the studies with no mismatch were more successful in finding a cognitive bias. These findings either indicate that the non-verbal cognitive bias tests taken as a starting point here are not suitable for testing Eldar et al. (2016)’s hypothesis or that the theory developed by Eldar et al. (2016) does not hold-up, at least in the settings used. For example, it has been shown that a single highly arousing negative event could, by itself, induces a change in the cognitive bias test (see Daros et al., 2014, calves dehorning; Lee et al., 2014, restrain and isolation stressor treatement in sheep). Whether such a change is actually a change in mood or a more temporary change must remain open. Mendl et al. (2010b) also argued that mood states are likely to reflect the cumulative experience of shorter term emotional episodes. Moreover, Bethell (2015) in her review supported the notion that experiments using longer-term affect manipulations showed mood-congruent shifts in judgment bias, too. Therefore, it is more plausible that the assessment of possible mismatches was too coarse given the highly variable approaches used for mood induction. In some instances, the actual occurrence of mismatches was difficult to be certain about in particular for individuals with a ‘history of poor welfare’ and ‘genetic animal model of depression’ that suggest no recent change in conditions but are likely to be a fairly long-lasting feature of each animal (e.g., personality). In these long-term situations, mismatches may no longer be relevant because what were mismatches initially have become the common situation. Also, we could not be sure how long ‘anxiety behavior’ had already been shown by animals before the studies were conducted. Moreover, the time between putative mood induction mismatch and cognitive judgment bias testing was not always known and variable. Indeed, we do not know at present how long into the future a mismatch is likely to exert its effect and how quickly repeated mismatches become a novel situation that is considered common. Therefore, we re-evaluated our model omitting studies including such long-term conditions (i.e., 24 independent tests from 18 studies) and we found that the general pattern remained the same (see Supplementary Figure S1). The corresponding *p*-value for mood induction mismatch dropped (χ^2^_2_ = 7.34, *p* = 0.026) indicating more strongly that, contrary to our expectation, the outcome of the studies with no mood induction mismatch were more likely to be as expected. This relatively low *p*-value could also be just the result of conducting an additional test (multiple testing issue). In general, most of the studies included in our data set did not specifically focus on creating cumulative mismatch in their approach and were therefore more pragmatic than theory-guided. In further studies, we advise to aim at creating cumulative expectation mismatch while inducing mood states to explicitly test the theory by Eldar et al. (2016).

We also expected that the success in mood induction may be modulated if the cognitive bias testing itself produced some short-term mismatch. The effects of the testing mismatch were evaluated in those studies only that had a negative mood induction mismatch because other combinations were observed rarely. We can therefore not exclude that testing mismatch would have a different effect after neutral or positive mood induction mismatch, so this does not seem likely. Additionally, there was some difficulty to assess the testing mismatches. We cannot negate the possibility that some testing mismatches occurred in additional studies but were not noted by neither the original authors nor us. These studies would then have been misclassified. Nevertheless, we found a strong pattern for the influence of the testing mismatch after a negative mood induction mismatch that would indicate an even stronger pattern given some risk of misclassification. Our results show that testing mismatch not only modulates the effect of mood induction mismatch (at least for negative mood induction mismatch, *n* = 7) but can even reverse that effect (at least for positive testing mismatch, *n* = 6). For four studies (Doyle et al., 2010; Briefer Freymond et al., 2014; Wheeler et al., 2015; Horváth et al., 2016), this positive testing mismatch corresponds to the notion of Baciadonna and McElligott (2015) that releasing animals from a short-term stressor induces positive emotional states. Doyle et al. (2010) explained that restrained and isolated subjects had a more positive outcome in the judgment task, either because the releasing from a restraining situation induced a more positive emotional state than with the unrestrained control subjects, or because the exposure to a strong negative treatment may have altered their risk-taking threshold. For two additional studies (Brydges et al., 2012; Parker et al., 2014), the positive testing mismatch seems to reflect a stimulating effect of the test that was stronger than the mood induction condition. The tests with a negative mood induction mismatch include two studies in which changes occurred during the test (i.e., unexpected decrease in confort during the test, Burman et al., 2009; Boleij et al., 2012) and three studies in which a learning effect about the unrewarded ambiguous probes was developed (i.e., the ambiguous cues became negative, Brilot et al., 2010; Doyle et al., 2011b; Murphy et al., 2013). As emphasized by Brilot et al. (2010) and Perdue (2017), subjects may rapidly learn the meaning of unrewarded ambiguous stimuli and this is reflected by reducing their responding rate or latency to respond, rather than being a negative shift in affective state. All these observations illustrate how important the effect of the testing mismatch could be. It is therefore crucial that this type of mismatch is carefully accounted for in the conduction of future cognitive bias tests.

In any type of meta-analysis, reporting bias can be a problem (Hooijmans et al., 2014). Here, this problem may at least have been less important given the fact that, overall, 38.89 % of the tests included in the study sample resulted in a failure to find a difference in cognitive bias.

## Conclusion

Given the low support found for the cumulative mismatch theory of mood based on the so far unsystematically conducted non-verbal cognitive bias studies, we advise to aim at creating cumulative expectation mismatch while inducing mood states. Furthermore, testing mismatch should be avoided as much as possible, because it might reverse the affective state of animals as measured in a cognitive judgment bias paradigm.

## Author Contributions

CR and LG made substantial contributions to the conception and design of the work. CR and JM participated in the acquisition of the data. CR and LG in the analysis and the interpretation of the data for the work. CR, JM, and LG participated in drafting the work or revising it critically for important intellectual content. CR, JM, and LG gave their final approval of the version to be published; and agree to be accountable for all aspects of the work.

## Conflict of Interest Statement

The authors declare that the research was conducted in the absence of any commercial or financial relationships that could be construed as a potential conflict of interest.

## Footnotes

1 http://www.webofknowledge.com
