# Supplementary material for: Mood As Cumulative Expectation Mismatch: A Test of Theory Based on Data from Non-verbal Cognitive Bias Tests
Source: Front Psychol. 2017 Dec 14;8:2197. doi: 10.3389/fpsyg.2017.02197 (PMC5824615; doi:10.3389/fpsyg.2017.02197)
Supplement: Supplementary file 1 [file Table_1.pdf]

Supplementary Material

Mood as cumulative expectation mismatch: a test of theory based on data from nonverbal cognitive bias tests

Camille M. C. Raoult, Julia Moser, Lorenz Gygax*

*** Correspondence:** Corresponding Author: lorenz.gygax@hu-berlin.de

1 Supplementary Table

Supplementary Table 1. Assessment of mismatches in non-verbal cognitive judgement bias paradigm studies published, or in press and available online, up to July 31st, 2017. These 95 cognitive bias studies include 162 independent cognitive bias tests.

CBT: cognitive bias test outcome (-: contrary to the hypothesis, 0: none, +: fitting with the hypothesis), MIM: mood induction mismatch score (-: negative, 0: none, +: positive), TM: testing mismatch score (-: negative, 0: none, +: positive), E: test excluded from the re-evaluation.

Reference	Species	CBT	MIM	TM	E
Harding et al. (2004)	Rat	+	-	0	
Bateson and Matheson (2007)	Starling	+	-	0	
Burman et al. (2008)	Rat	+	-	-	
Burman et al. (2008)	Rat	0	-	0	
Matheson et al. (2008)	Starling	+	-	0	
Burman et al. (2009)	Rat	+	-	-	
Brilot et al. (2010)	Starling	0	-	-	
Doyle et al. (2010)	Sheep	-	-	+	
Enkel et al. (2010)	Rat	+	-	0	x
Enkel et al. (2010)	Rat	+	-	0	
Mendl et al. (2010)	Dog	+	0	0	
Bateson et al. (2011)	Bee	+	0	0	
Brydges et al. (2011)	Rat	+	+	0	
Burman et al. (2011)	Dog	-	0	-	
Doyle et al. (2011a)	Sheep	+	-	0	
Doyle et al. (2011b)	Sheep	+	-	-	
Salmeto et al. (2011)	Chicken	+	0	0	
Sanger et al. (2011)	Sheep	+	0	0	
Anderson et al. (2012)	Human	+	-	0	
Bethell et al. (2012)	Rhesus	+	-	0	
Boleij et al. (2012)	Mice	+	-	0	
Boleij et al. (2012)	Mice	+	-	-	x
Brydges et al. (2012)	Rat	-	-	+	
Destrez et al. (2012)	Sheep	+	+	0	
Douglas et al. (2012)	Pig	+	-	0	
Douglas et al. (2012)	Pig	+	-	0	

Reference	Species	CBT	MIM	TM	E
Douglas et al. (2012)	Pig	+	-	0	
Muller et al. (2012)	Dog	0	0	-	
Pomerantz et al. (2012)	Capuchin	+	-	0	x
Pomerantz et al. (2012)	Capuchin	0	-	0	x
Richter et al. (2012)	Rat	+	-	0	x
Richter et al. (2012)	Rat	+	0	0	
Rygula et al. (2012)	Rat	+	0	0	
Wichman et al. (2012)	Chicken	0	+	0	
Anderson et al. (2013)	Rat	0	+	0	
Anderson et al. (2013)	Rat	0	+	-	
Anderson et al. (2013)	Rat	0	+	0	
Anderson et al. (2013)	Rat	0	+	0	
Briefer and McElligott (2013)	Goat	-	0	0	x
Briefer and McElligott (2013)	Goat	0	0	0	x
Chaby et al. (2013)	Rat	+	-	0	
Destrez et al. (2013)	Sheep	+	-	0	
Düpjan et al. (2013)	Pig	0	-	0	
Murphy et al. (2013)	Pig	0	-	0	x
Murphy et al. (2013)	Pig	0	-	-	
Neave et al. (2013)	Calve	+	0	0	
Papciak et al. (2013)	Rat	+	-	0	
Rygula et al. (2013)	Rat	+	-	0	
Schick et al. (2013)	Human	+	-	0	
Seehuus et al. (2013)	Chicken	+	-	0	
Titulaer et al. (2013)	Dog	0	-	0	
Briefer Freymond et al. (2014)	Horse	-	-	+	
Daros et al. (2014)	Cow	+	-	0	
Daros et al. (2014)	Cow	+	0	0	
Destrez et al. (2014)	Sheep	+	-	0	
Keen et al. (2014)	Bear	0	+	0	
Keen et al. (2014)	Bear	0	+	0	
Kloke et al. (2014)	Mice	+	-	0	
Kloke et al. (2014)	Mice	0	-	0	x
Lansade et al. (2014)	Horse	+	+	0	
Parker et al. (2014)	Rat	-	-	+	
Rygula et al. (2014a)	Rat	+	+	0	
Rygula et al. (2014a)	Rat	+	+	0	
Rygula et al. (2014a)	Rat	+	+	0	
Rygula et al. (2014b)	Rat	0	+	0	
Rygula et al. (2014b)	Rat	-	+	0	
Scollo et al. (2014)	Pig	0	-	-	
Verbeek et al. (2014a)	Sheep	+	-	0	
Verbeek et al. (2014a)	Sheep	+	-	0	
Verbeek et al. (2014b)	Sheep	+	+	0	
Verbeek et al. (2014b)	Sheep	+	-	0	
Vögeli et al. (2014)	Sheep	0	-	0	

**Reference**	**Species**	**CBT**	**MIM**	**TM**	**E**
Walker et al. (2014)	Dog	0	-	0	
Bateson et al. (2015)	Starling	+	-	0	
Bethell and Koyama (2015)	Hamster	+	-	0	
Brajon et al. (2015)	Pig	+	-	0	
Brajon et al. (2015)	Pig	+	+	0	
Coulon et al. (2015)	Sheep	+	-	0	
Coulon et al. (2015)	Sheep	0	-	0	x
da Cunha Nogueira et al. (2015)	Peccary	+	-	0	
Gordon and Rogers (2015)	Marmoset	+	-	0	x
Guldimann et al. (2015)	Sheep	0	-	0	
Hernandez et al. (2015)	Hens	+	0	0	
Karagiannis et al. (2015)	Dog	+	-	0	
Karagiannis et al. (2015)	Dog	+	0	0	
Kis et al. (2015)	Dog	+	+	0	
McGuire et al. (2015)	Rat	0	+	+	
Murphy et al. (2015)	Pig	+	-	0	x
Rygula et al. (2015a)	Rat	0	+	0	
Rygula et al. (2015a)	Rat	0	+	0	
Rygula et al. (2015a)	Rat	+	+	0	
Rygula et al. (2015a)	Rat	0	+	0	
Rygula et al. (2015b)	Rat	0	+	0	
Rygula et al. (2015b)	Rat	0	+	0	
Wheeler et al. (2015)	Rat	+	+	0	
Wheeler et al. (2015)	Rat	+	-	0	
Wheeler et al. (2015)	Rat	+	-	+	
Wheeler et al. (2015)	Rat	+	+	-	
Ash and Buchanan-Smith (2016)	Marmoset	0	-	0	x
Ash and Buchanan-Smith (2016)	Marmoset	0	-	0	x
Asher et al. (2016)	Pig	+	-	0	x
Asher et al. (2016)	Pig	+	+	0	
Baciadonna et al. (2016)	Goat	0	+	0	
Barker et al. (2016)	Rat	+	-	0	
Barker et al. (2016)	Rat	0	-	0	
Brown et al. (2016)	Rat	+	0	0	x
Carreras et al. (2016a)	Pig	0	-	0	x
Carreras et al. (2016a)	Pig	+	-	0	x
Carreras et al. (2016b)	Pig	0	-	0	
Carreras et al. (2016b)	Pig	0	-	0	
Deakin et al. (2016)	Hen	+	0	+	
Horváth et al. (2016)	Quail	0	+	+	
Horváth et al. (2016)	Quail	0	-	+	
Horváth et al. (2016)	Quail	+	+	0	
Horváth et al. (2016)	Quail	0	-	0	
Horváth et al. (2016)	Quail	0	-	0	
Horváth et al. (2016)	Quail	0	+	0	
Horváth et al. (2016)	Quail	0	-	0	

Reference	Species	CBT	MIM	TM	E
Horváth et al. (2016)	Quail	0	-	0	
Horváth et al. (2016)	Quail	0	-	0	
Horváth et al. (2016)	Quail	0	+	0	
Horváth et al. (2016)	Quail	0	-	0	
Kasbaoui et al. (2016)	Cat	0	-	0	
Kregiel et al. (2016)	Rat	0	+	0	
Kregiel et al. (2016)	Rat	0	+	0	
Kregiel et al. (2016)	Rat	+	+	0	
Kregiel et al. (2016)	Rat	+	+	0	
Kregiel et al. (2016)	Rat	0	-	0	
Kregiel et al. (2016)	Rat	0	-	0	
Kregiel et al. (2016)	Rat	0	-	0	
Kregiel et al. (2016)	Rat	0	-	0	
Löckener et al. (2016)	Horse	+	-	0	
Löckener et al. (2016)	Horse	+	+	0	
Novak et al. (2016a)	Mice	+	-	0	x
Novak et al. (2016a)	Mice	0	-	0	x
Novak et al. (2016a)	Mice	0	-	0	x
Novak et al. (2016b)	Mice	+	-	0	
Oliveira et al. (2016)	Peccary	+	-	0	
Oliveira et al. (2016)	Peccary	-	0	-	
Oliveira et al. (2016)	Peccary	0	0	0	
Perry et al. (2016)	Bee	+	0	0	
Perry et al. (2016)	Bee	+	-	0	
Schino et al. (2016)	Monkey	0	0	0	
Schino et al. (2016)	Monkey	+	+	0	
Barker et al. (2017)	Rat	+	-	0	
Barker et al. (2017)	Rat	-	0	0	x
Destrez et al. (2017)	Sheep	+	-	0	
d'Ettorre et al. (2017)	Ant	+	-	0	x
Golebiowska and Rygula (2017a)	Rat	-	+	0	
Golebiowska and Rygula (2017a)	Rat	0	-	0	
Golebiowska and Rygula (2017a)	Rat	0	+	0	
Golebiowska and Rygula (2017b)	Rat	+	+	0	
Golebiowska and Rygula (2017b)	Rat	0	+	0	
Henry et al. (2017)	Horse	+	-	0	
Lalot et al. (2017)	Canary	+	+	0	
Lalot et al. (2017)	Canary	+	+	0	
Lalot et al. (2017)	Canary	+	-	0	
Roelofs et al. (2017)	Pig	0	0	-	x
Schlüns et al. (2017)	Bee	+	0	0	
Schlüns et al. (2017)	Bee	0	-	0	
Schlüns et al. (2017)	Bee	0	-	0	
Stracke et al. (2017)	Pig	+	-	0	

Studies were identified, as in Gygax (2014), by searching the Web of Science (http://www.webofknowledge.com) until July 31st 2017 using a cited-reference search for

‘Harding et al. 2004’ combined with (OR) the key word combination ‘((cognitive AND bias AND welfare) OR (judgement AND bias AND welfare))’. We used an additional search consisting of a cited-reference search for ‘Harding et al. 2004’ combined with (AND) the key word combination ‘((cognitive AND bias) OR (judgement AND bias))’, a third search with the key word combination ‘(affective AND state) AND ((cognitive AND bias) OR (judgement AND bias))’, and a forth search with the key word combination ‘(state AND affect) AND ((cognitive AND bias) OR (judgement AND bias))’. We also conducted the latter search using ‘(affective AND state) *OR* ((cognitive AND bias) OR (judgement AND bias))’. With this search, > 20’000 hits were reached. The first dozens of hits were irrelevant to a large extent and therefore, we did not further pursue this line of search. Only studies that attempted to actively induce mood changes or in which mood was independently inferred (e.g., based on self-reports, behavioral, or physiological data) were included. Other methodological studies were excluded from this analysis, as well as publications including fewer than 4 subjects (per group treatment), because they are lacking the necessary degrees of freedom for a quantitative statistical evaluation at group level.

2 Supplementary Figure

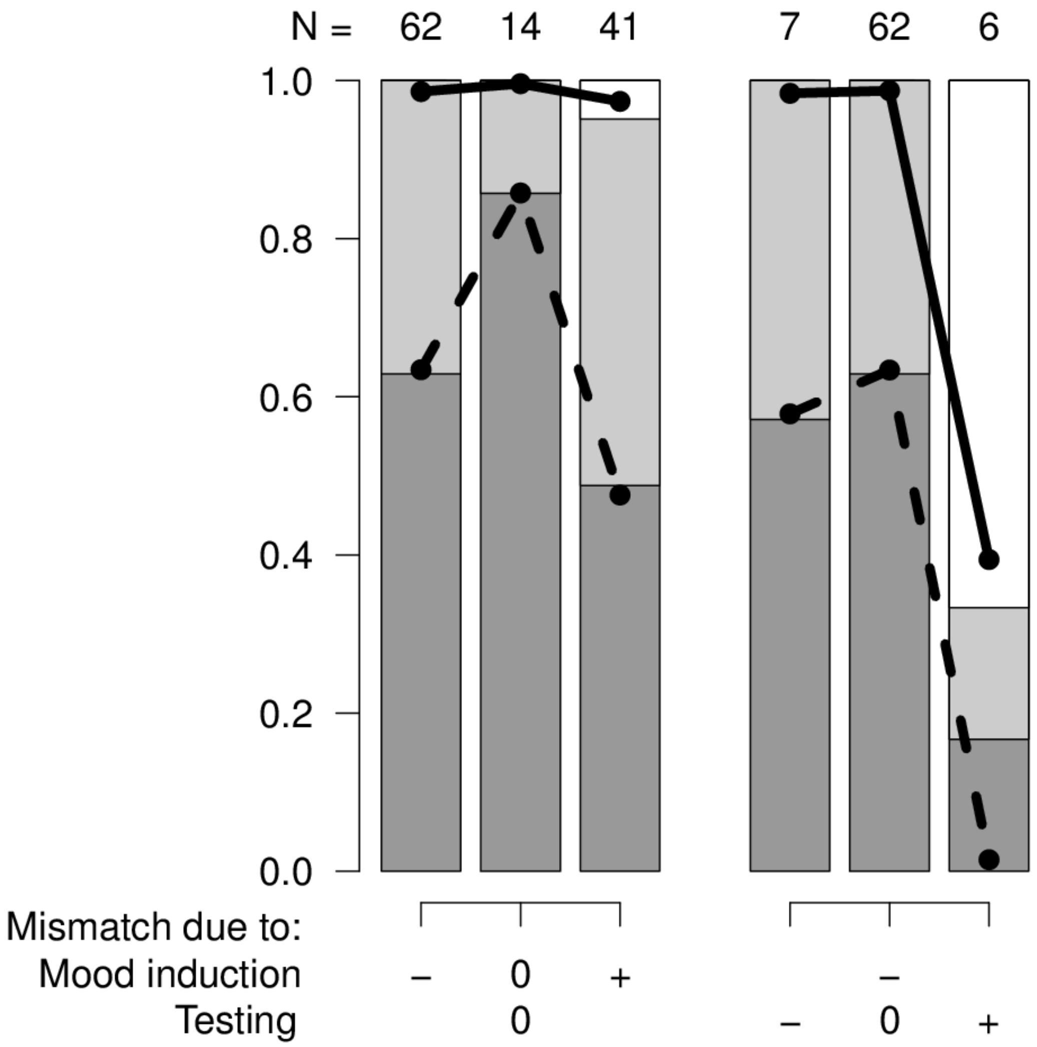

Supplementary Figure 1. Success of independent cognitive bias tests (dark grey: fitting with the hypothesis, light grey: none, white: contrary to the hypothesis) in function of the mood induction mismatch (-: negative, 0: none, +: positive) and the testing mismatch (-: negative, 0: none, +: positive) for the re-evaluation of the model omitting studies including long-term conditions which are unlikely to cause a recent change in mood (i.e., 24 independent tests from 18 studies, see Supplementary Table 1). In the studies without testing mismatch, there was a slightly higher proportion of successful cognitive bias studies when there was no cumulative mood induction mismatch compared with either a positive or a negative mismatch, which was supported by a moderately low p-value (χ^2_2 = 7.34, *P* = 0.026). With a negative cumulative mood induction mismatch, the success of cognitive bias studies decreased from no testing mismatch and a negative testing mismatch to a positive testing mismatch (χ^2_2 = 17.91, *P* = 0.0001). N: number of independent tests in each combination. Black lines: model estimates that reflect the probability of the switches from one level in the outcome variable to the next.

3 Study references

Anderson, M.H., Hardcastle, C., Munafo, M.R., and Robinson, E.S. (2012). Evaluation of a novel translational task for assessing emotional biases in different species. *Cognitive, Affective & Behavioral Neuroscience* 12(2)**,** 373-381. doi: 10.3758/s13415-011-0076-4.

Anderson, M.H., Munafo, M.R., and Robinson, E.S. (2013). Investigating the psychopharmacology of cognitive affective bias in rats using an affective tone discrimination task. *Psychopharmacology* 226(3)**,** 601-613. doi: 10.1007/s00213-012-2932-5.

Ash, H., and Buchanan-Smith, H.M. (2016). The long-term impact of infant rearing background on the affective state of adult common marmosets (Callithrix jacchus). *Applied Animal Behaviour Science* 174**,** 128-136. doi: 10.1016/j.applanim.2015.10.009.

Asher, L., Friel, M., Griffin, K., and Collins, L.M. (2016). Mood and personality interact to determine cognitive biases in pigs. *Biology Letters* 12**,** 20160402. doi: 10.1098/rsbl.2016.0402.

Baciadonna, L., Nawroth, C., and McElligott, A.G. (2016). Judgement bias in goats (Capra hircus) investigating the effects of human grooming. *Peer J* 4**,** e2485. doi: 10.7717/peerj.2485.

Barker, T.H., Bobrovskaya, G.S., Howarth, G.S., and Whittaker, A.L. (2017). Female rats display fewer optimistic responses in a judgment bias test in the absence of a physiological stress response. *Physiology & Behavior* 173**,** 124-131. doi: 10.1016/j.physbeh.2017.02.006.

Barker, T.H., Howarth, G.S., and Whittaker, A.L. (2016). The effects of metabolic cage housing and sex on cognitive bias expression in rats. *Applied Animal Behaviour Science* 177**,** 70-76. doi: 10.1016/j.applanim.2016.01.018.

Bateson, M., Desire, S., Gartside, S.E., and Wright, G.A. (2011). Agitated honeybees exhibit pessimistic cognitive biases. *Current Biology* 21(12)**,** 1070-1073. doi: 10.1016/j.cub.2011.05.017.

Bateson, M., Emmerson, M., Ergun, G., Monaghan, P., and Nettle, D. (2015). Opposite Effects of Early-Life Competition and Developmental Telomere Attrition on Cognitive Biases in Juvenile European Starlings. *PLoS One* 10(7)**,** e0132602. doi: 10.1371/journal.pone.0132602.

Bateson, M., and Matheson, S.M. (2007). Performance on a categorisation task suggests that removal of environmental enrichment induces 'pessimism' in captive European starlings (Sturnus vulgaris). *Animal Welfare* 16(Suppl)**,** 33-36.

Bethell, E.J., Holmes, A., MacLarnon, A., and Semple, S. (2012). Cognitive bias in a non-human primate: husbandry procedures influence cognitive indicators of psychological well-being in captive rhesus macaques. *Animal Welfare* 21**,** 185-195. doi: 10.7120/09627286.21.2.185.

Bethell, E.J., and Koyama, N.F. (2015). Happy hamsters? Enrichment induces positive judgement bias for mildly (but not truly) ambiguous cues to reward and punishment in Mesocricetus auratus. *Royal Society Open Science* 2(7)**,** 140399. doi: 10.1098/rsos.140399.

Boleij, H., van't Klooster, J., Lavrijsen, M., Kirchhoff, S., Arndt, S.S., and Ohl, F. (2012). A test to identify judgement bias in mice. *Behavioural Brain Research* 233(1)**,** 45-54. doi: 10.1016/j.bbr.2012.04.039.

Brajon, S., Laforest, J.P., Schmitt, O., and Devillers, N. (2015). The Way Humans Behave Modulates the Emotional State of Piglets. *PLoS One* 10(8)**,** e0133408. doi: 10.1371/journal.pone.0133408.

Briefer, E.F., and McElligott, A.G. (2013). Rescued goats at a sanctuary display positive mood after former neglect. *Applied Animal Behaviour Science* 146**,** 45-55. doi: 10.1016/j.applanim.2013.03.007.

Briefer Freymond, S., Briefer, E.F., Zollinger, A., Gindrat-von Allmen, Y., Wyss, C., and Bachmann, I. (2014). Behaviour of horses in a judgment bias test associated with positive or

negative reinforcement. *Applied Animal Behaviour Science* 158**,** 34-45. doi: 10.1016/j.applanim.2014.06.006.

Brilot, B.O., Asher, L., and Bateson, M. (2010). Stereotyping starlings are more 'pessimistic'. *Animal Cognition* 13(5)**,** 721-731. doi: 10.1007/s10071-010-0323-z.

Brown, G.R., Cullum, P., Martin, S., and Healy, S.D. (2016). Sex differences in performance on a cognitive bias task in Norway rats. *Behavioural Processes* 133**,** 52-55. doi: 10.1016/j.beproc.2016.11.005.

Brydges, N.M., Hall, L., Nicolson, R., Holmes, M.C., and Hall, J. (2012). The effects of juvenile stress on anxiety, cognitive bias and decision making in adulthood: a rat model. *PLoS One* 7(10)**,** e48143. doi: 10.1371/journal.pone.0048143.

Brydges, N.M., Leach, M., Nicol, K., Wright, R., and Bateson, M. (2011). Environmental enrichment induces optimistic cognitive bias in rats. *Animal Behaviour* 81**,** 169-175. doi: 10.1016/j.anbehav.2010.09.030.

Burman, O.H., McGowan, R., Mendl, M., Norling, Y., Paul, E., Rehn, T., et al. (2011). Using judgement bias to measure positive affective state in dogs. *Applied Animal Behaviour Science* 132**,** 160-168. doi: 10.1016/j.applanim.2011.04.001.

Burman, O.H., Parker, R.M., Paul, E.S., and Mendl, M. (2008). A spatial judgement task to determine background emotional state in laboratory rats, Rattus norvegicus. *Animal Behaviour* 76(3)**,** 801-809. doi: 10.1016/j.anbehav.2008.02.014.

Burman, O.H., Parker, R.M., Paul, E.S., and Mendl, M.T. (2009). Anxiety-induced cognitive bias in non-human animals. *Physioly & Behavior* 98(3)**,** 345-350. doi: 10.1016/j.physbeh.2009.06.012.

Carreras, R., Arroyo, L., Mainau, E., Pena, R., Bassols, A., Dalmau, A., et al. (2016a). Effect of gender and halothane genotype on cognitive bias and its relationship with fear in pigs. *Applied Animal Behaviour Science* 177**,** 12-18. doi: 10.1016/j.applanim.2016.01.019.

Carreras, R., Mainau, E., Arroyo, L., Moles, X., González, J., Bassols, A., et al. (2016b). Housing conditions do not alter cognitive bias but affect serum cortisol, qualitative behaviour assessment and wounds on the carcass in pigs. *Applied Animal Behaviour Science* 185**,** 39–44. doi: 10.1016/j.applanim.2016.09.006.

Chaby, L.E., Cavigelli, S.A., White, A., Wang, K., and Braithwaite, V.A. (2013). Long-term changes in cognitive bias and coping response as a result of chronic unpredictable stress during adolescence. *Frontiers in Human Neuroscience* 7**,** 328. doi: 10.3389/fnhum.2013.00328.

Coulon, M., Nowak, R., Andanson, S., Petit, B., Levy, F., and Boissy, A. (2015). Effects of prenatal stress and emotional reactivity of the mother on emotional and cognitive abilities in lambs. *Developmental Psychobiology* 57(5)**,** 626-636. doi: 10.1002/dev.21320.

d'Ettorre, P., Carere, C., Demora, L., Le Quinquis, P., Signorotti, L., and Bovet, D. (2017). Individual differences in exploratory activity relate to cognitive judgement bias in carpenter ants. *Behavioural Processes* 134**,** 63-69. doi: 10.1016/j.beproc.2016.09.008.

da Cunha Nogueira, S.S., Fernandes, I.K., Costa, T.S., Nogueira-Filho, S.L., and Mendl, M. (2015). Does Trapping Influence Decision-Making under Ambiguity in White-Lipped Peccary (Tayassu pecari)? *PLoS One* 10(6)**,** e0127868. doi: 10.1371/journal.pone.0127868.

Daros, R.R., Costa, J.H., von Keyserlingk, M.A., Hotzel, M.J., and Weary, D.M. (2014). Separation from the dam causes negative judgement bias in dairy calves. *PLoS One* 9(5)**,** e98429. doi: 10.1371/journal.pone.0098429.

Deakin, A., Browne, W.J., Hodge, J.J.L., Paul, E., and Mendl, M. (2016). A screen-peck task for investigating cognitive bias in laying hens. *PLoS One* 11(7)**,** 1-13. doi: 10.1371/journal.

Destrez, A., Boissy, A., Guilloteau, L., Andanson, S., Souriau, A., Laroucau, K., et al. (2017). Effects of a chronic stress treatment on vaccinal response in lambs. *Animal* 11(5)**,** 872-880. doi: 10.1017/S1751731116002317.

Destrez, A., Deiss, V., Belzung, C., Lee, C., and Boissy, A. (2012). Does reduction of fearfulness tend to reduce pessimistic-like judgment in lambs? *Applied Animal Behaviour Science* 139**,** 233-241. doi: 10.1016/j.applanim.2012.04.006.

Destrez, A., Deiss, V., Leterrier, C., Calandreau, L., and Boissy, A. (2014). Repeated exposure to positive events induces optimistic-like judgment and enhances fearfulness in chronically stressed sheep. *Applied Animal Behaviour Science* 154**,** 30-38. doi: 10.1016/j.applanim.2014.01.005.

Destrez, A., Deiss, V., Lévy, F., Calandreau, L., Lee, C., Challou-Sagon, E., et al. (2013). Chronic stress induces pessimistic-like judgment and learning deficits in sheep. *Applied Animal Behaviour Science* 148**,** 28-36. doi: 10.1016/j.applanim.2013.07.016.

Douglas, C., Bateson, M., Walsh, C., Bédué, A., and Edwards, S.A. (2012). Environmental enrichment induces optimistic cognitive biases in pigs. *Applied Animal Behaviour Science* 139**,** 65-73. doi: 10.1016/j.applanim.2012.02.018.

Doyle, R.E., Fisher, A.D., Hinch, G.N., Boissy, A., and Lee, C. (2010). Release from restraint generates a positive judgement bias in sheep. *Applied Animal Behaviour Science* 122**,** 28-34. doi: 10.1016/j.applanim.2009.11.003.

Doyle, R.E., Hinch, G.N., Fisher, A.D., Boissy, A., Henshall, J.M., and Lee, C. (2011a). Administration of serotonin inhibitor p-Chlorophenylalanine induces pessimistic-like judgement bias in sheep. *Psychoneuroendocrinology* 36(2)**,** 279-288. doi: 10.1016/j.psyneuen.2010.07.018.

Doyle, R.E., Lee, C., Deiss, V., Fisher, A.D., Hinch, G.N., and Boissy, A. (2011b). Measuring judgement bias and emotional reactivity in sheep following long-term exposure to unpredictable and aversive events. *Physioly & Behavior* 102(5)**,** 503-510. doi: 10.1016/j.physbeh.2011.01.001.

Düpjan, S., Ramp, C., Kanitz, E., Tuchscherer, A., and Puppe, B. (2013). A design for studies on cognitive bias in the domestic pig. *Journal of Veterinary Behavior* 8**,** 485-489. doi: 10.1016/j.jveb.2013.05.007.

Enkel, T., Gholizadeh, D., von Bohlen Und Halbach, O., Sanchis-Segura, C., Hurlemann, R., Spanagel, R., et al. (2010). Ambiguous-cue interpretation is biased under stress- and depression-like states in rats. *Neuropsychopharmacology* 35(4)**,** 1008-1015. doi: 10.1038/npp.2009.204.

Golebiowska, J., and Rygula, R. (2017a). Effects of acute dopaminergic and serotonergic manipulations in the ACI paradigm depend on the basal valence of cognitive judgement bias in rats. *Behavioural Brain Research* 327**,** 133-143. doi: 10.1016/j.bbr.2017.02.013.

Golebiowska, J., and Rygula, R. (2017b). Lesions of the Orbitofrontal but Not Medial Prefrontal Cortex Affect Cognitive Judgment Bias in Rats. *Frontiers in Behavioral Neuroscience* 11(51)**,** 1-9. doi: 10.3389/fnbeh.2017.00051.

Gordon, D.J., and Rogers, L.J. (2015). Cognitive bias, hand preference and welfare of common marmosets. *Behavioural Brain Research* 287**,** 100-108. doi: 10.1016/j.bbr.2015.03.037.

Guldimann, K., Vögeli, S., Wolf, M., Wechsler, B., and Gygax, L. (2015). Frontal brain deactivation during a non-verbal cognitive judgement bias test in sheep. *Brain and Cognition* 93**,** 35-41. doi: 10.1016/j.bandc.2014.11.004.

Gygax, L. (2014). The A to Z of statistics for testing cognitive judgement bias. *Animal Behaviour* 95**,** 59-69. doi: 10.1016/j.anbehav.2014.06.013.

Harding, E.J., Paul, E.S., and Mendl, M. (2004). Cognitive bias and affective state. *Nature Publishing Group* 427**,** 312.

Henry, S., Fureix, C., Rowberry, R., Bateson, M., and Hausberger, M. (2017). Do horses with poor welfare show 'pessimistic' cognitive biases? *The Science of Nature* 104(8)**,** 1-15. doi: 10.1007/s00114-016-1429-1.

Hernandez, C.E., Hinch, G.N., Lea, J., Ferguson, D., and Lee, C. (2015). Acute stress enhances sensitivity to a highly attractive food reward without affecting judgement bias in laying hens. *Applied Animal Behaviour Science* 163**,** 135-143. doi: 10.1016/j.applanim.2014.12.002.

Horváth, M., Pichová, K., and Kost'ál, L. (2016). The effects of housing conditions on judgement bias in Japanese quail. *Applied Animal Behaviour Science* 185**,** 121-130. doi: 10.1016/j.applanim.2016.09.007.

Karagiannis, C.I., Burman, O.H., and Mills, D.S. (2015). Dogs with separation-related problems show a "less pessimistic" cognitive bias during treatment with fluoxetine (Reconcile) and a behaviour modification plan. *BMC Veterinary Research* 11**,** 80. doi: 10.1186/s12917-015-0373-1.

Kasbaoui, N., Cooper, J., Mills, D.S., and Burman, O. (2016). Effects of Long-Term Exposure to an Electronic Containment System on the Behaviour and Welfare of Domestic Cats. *PLoS One* 11(9)**,** 1-20. doi: 10.1371/journal.pone.0162073.

Keen, H.A., Nelson, O.L., Robbins, C.T., Evans, M., Shepherdson, D.J., and Newberry, R.C. (2014). Validation of a novel cognitive bias task based on difference in quantity of reinforcement for assessing environmental enrichment. *Animal Cognition* 17(3)**,** 529-541. doi: 10.1007/s10071-013-0684-1.

Kis, A., Hernadi, A., Kanizsar, O., Gacsi, M., and Topal, J. (2015). Oxytocin induces positive expectations about ambivalent stimuli (cognitive bias) in dogs. *Hormones and Behavior* 69**,** 1-7. doi: 10.1016/j.yhbeh.2014.12.004.

Kloke, V., Schreiber, R.S., Bodden, C., Mollers, J., Ruhmann, H., Kaiser, S., et al. (2014). Hope for the best or prepare for the worst? Towards a spatial cognitive bias test for mice. *PLoS One* 9(8)**,** e105431. doi: 10.1371/journal.pone.0105431.

Kregiel, J., Malek, N., Popik, P., Starowicz, K., and Rygula, R. (2016). Anandamide mediates cognitive judgement bias in rats. *Neuropharmacology* 101**,** 146-153. doi: 10.1016/j.neuropharm.2015.09.009.

Lalot, M., Ung, D., Peron, F., d'Ettorre, P., and Bovet, D. (2017). You know what? I'm happy. Cognitive bias is not related to personality but is induced by pair-housing in canaries (Serinus canaria). *Behavioural Processes* 134**,** 70-77. doi: 10.1016/j.beproc.2016.09.012.

Lansade, L., Valenchon, M., Foury, A., Neveux, C., Cole, S.W., Laye, S., et al. (2014). Behavioral and Transcriptomic Fingerprints of an Enriched Environment in Horses (Equus caballus). *PLoS One* 9(12)**,** e114384. doi: 10.1371/journal.pone.0114384.

Löckener, S., Reese, S., Erhard, M., and Wöhr, A.-C. (2016). Pasturing in herds after housing in horseboxes induces a positive cognitive bias in horses. *Journal of Veterinary Behavior* 11**,** 50-55. doi: 10.1016/j.jveb.2015.11.005.

Matheson, S.M., Asher, L., and Bateson, M. (2008). Larger, enriched cages are associated with 'optimistic' response biases in captive European starlings (Sturnus vulgaris). *Applied Animal Behaviour Science* 109**,** 374-383. doi: 10.1016/j.applanim.2007.03.007.

McGuire, M.C., Williams, K.L., Welling, L.L., and Vonk, J. (2015). Cognitive bias in rats is not influenced by oxytocin. *Frontiers in Psychology* 6**,** 1306. doi: 10.3389/fpsyg.2015.01306.

Mendl, M., Brooks, J., Basse, C., Burman, O., Paul, E., Blackwell, E., et al. (2010). Dogs showing separation-related behaviour exhibit a 'pessimistic' cognitive bias. *Current Biology* 20(19)**,** R839-840. doi: 10.1016/j.cub.2010.08.030.

Muller, C.A., Riemer, S., Rosam, C.M., Schosswender, J., Range, F., and Huber, L. (2012). Brief owner absence does not induce negative judgement bias in pet dogs. *Animal Cognition* 15(5)**,** 1031-1035. doi: 10.1007/s10071-012-0526-6.

Murphy, E., Kraak, L., van den Broek, J., Nordquist, R.E., and van der Staay, F.J. (2015). Decision-making under risk and ambiguity in low-birth-weight pigs. *Animal Cognition* 18(2)**,** 561-572. doi: 10.1007/s10071-014-0825-1.

Murphy, E., Nordquist, R.E., and Van der Staay, F.J. (2013). Responses of conventional pigs and Göttingen miniature pigs in an active choice judgement bias task. *Applied Animal Behaviour Science* 148**,** 64-76. doi: 10.1016/j.applanim.2013.07.011.

Neave, H.W., Daros, R.R., Costa, J.H., von Keyserlingk, M.A., and Weary, D.M. (2013). Pain and pessimism: dairy calves exhibit negative judgement bias following hot-iron disbudding. *PLoS One* 8(12)**,** e80556. doi: 10.1371/journal.pone.0080556.

Novak, J., Bailoo, J.D., Melotti, L., and Wurbel, H. (2016a). Effect of Cage-Induced Stereotypies on Measures of Affective State and Recurrent Perseveration in CD-1 and C57BL/6 Mice. *PLoS One* 11(5)**,** e0153203. doi: 10.1371/journal.pone.0153203.

Novak, J., Stojanovski, K., Melotti, L., Reichlin, T.S., Palme, R., and Wurbel, H. (2016b). Effects of stereotypic behaviour and chronic mild stress on judgement bias in laboratory mice. *Applied Animal Behaviour Science* 174**,** 162-172. doi: 10.1016/j.applanim.2015.10.004.

Oliveira, F.R.M., Nogueira-Filho, S.L., Sousa, M.B.C., Dias, C.T.S., Mendl, M., and Nogueira, S.S. (2016). Measurement of cognitive bias and cortisol levels to evaluate the effects of space restriction on captive collared peccary (Mammalia, Tayassuidae). *Applied Animal Behaviour Science* 181**,** 76-82. doi: 10.1016/j.applanim.2016.05.021.

Papciak, J., Popik, P., Fuchs, E., and Rygula, R. (2013). Chronic psychosocial stress makes rats more 'pessimistic' in the ambiguous-cue interpretation paradigm. *Behavioural Brain Research* 256**,** 305-310. doi: 10.1016/j.bbr.2013.08.036.

Parker, R.M., Paul, E.S., Burman, O.H., Browne, W.J., and Mendl, M. (2014). Housing conditions affect rat responses to two types of ambiguity in a reward-reward discrimination cognitive bias task. *Behavioural Brain Research* 274**,** 73-83. doi: 10.1016/j.bbr.2014.07.048.

Perry, C.J., Baciadonna, L., and Chittka, L. (2016). Unexpected rewards induce dopamine-dependent positive emotion-like state changes in bumblebees. *Science* 353(6307)**,** 1529-1531. doi: 10.1126/science.aaf4454.

Pomerantz, O., Terkel, J., Suomi, S.J., and Paukner, A. (2012). Stereotypic head twirls, but not pacing, are related to a 'pessimistic'-like judgment bias among captive tufted capuchins (Cebus apella). *Animal Cognition* 15(4)**,** 689-698. doi: 10.1007/s10071-012-0497-7.

Richter, S.H., Schick, A., Hoyer, C., Lankisch, K., Gass, P., and Vollmayr, B. (2012). A glass full of optimism: enrichment effects on cognitive bias in a rat model of depression. *Cognitive, Affective & Behavioral Neuroscience* 12(3)**,** 527-542. doi: 10.3758/s13415-012-0101-2.

Roelofs, S., Nordquist, R.E., and van der Staay, F.Z. (2017). Female and male pigs' performance in a spatial holeboard and judgment bias task. *Applied Animal Behaviour Science* 191**,** 5-16. doi: 10.1016/j.applanim.2017.01.016.

Rygula, R., Golebiowska, J., Kregiel, J., Holuj, M., and Popik, P. (2015a). Acute administration of lithium, but not valproate, modulates cognitive judgment bias in rats. *Psychopharmacology* 232(12)**,** 2149-2156. doi: 10.1007/s00213-014-3847-0.

Rygula, R., Papciak, J., and Popik, P. (2013). Trait pessimism predicts vulnerability to stress-induced anhedonia in rats. *Neuropsychopharmacology* 38(11)**,** 2188-2196. doi: 10.1038/npp.2013.116.

Rygula, R., Papciak, J., and Popik, P. (2014a). The effects of acute pharmacological stimulation of the 5-HT, NA and DA systems on the cognitive judgement bias of rats in the ambiguous-cue interpretation paradigm. *European Neuropsychopharmacology* 24(7)**,** 1103-1111. doi: 10.1016/j.euroneuro.2014.01.012.

Rygula, R., Pluta, H., and Popik, P. (2012). Laughing rats are optimistic. *PLoS One* 7(12)**,** e51959. doi: 10.1371/journal.pone.0051959.

Rygula, R., Szczech, E., Kregiel, J., Golebiowska, J., Kubik, J., and Popik, P. (2015b). Cognitive judgment bias in the psychostimulant-induced model of mania in rats. *Psychopharmacology* 232**,** 651-660. doi: 10.1007/s00213-014-3707-y.

Rygula, R., Szczech, E., Papciak, J., Nikiforuk, A., and Popik, P. (2014b). The effects of cocaine and mazindol on the cognitive judgement bias of rats in the ambiguous-cue interpretation paradigm. *Behavioural Brain Research* 270**,** 206-212. doi: 10.1016/j.bbr.2014.05.026.

Salmeto, A.L., Hymel, K.A., Carpenter, E.C., Brilot, B.O., Bateson, M., and Sufka, K.J. (2011). Cognitive bias in the chick anxiety-depression model. *Brain Research* 1373**,** 124-130. doi: 10.1016/j.brainres.2010.12.007.

Sanger, M.E., Doyle, R.E., Hinch, G.N., and Lee, C. (2011). Sheep exhibit a positive judgement bias and stress-induced hyperthermia following shearing. *Applied Animal Behaviour Science* 131**,** 94-103. doi: 10.1016/j.applanim.2011.02.001.

Schick, A., Wessa, M., Vollmayr, B., Kuehner, C., and Kanske, P. (2013). Indirect assessment of an interpretation bias in humans: neurophysiological and behavioral correlates. *Frontiers in Human Neuroscience* 7**,** 272. doi: 10.3389/fnhum.2013.00272.

Schino, G., Massimei, R., Pinzaglia, M., and Addessi, E. (2016). Grooming, social rank and 'optimism' in tufted capuchin monkeys: a study of judgement bias. *Animal Behaviour* 119**,** 11-16. doi: 10.1016/j.anbehav.2016.06.017.

Schlüns, H., Wellinh, H., Federici, J.R., and Lewejohann, L. (2017). The glass is not yet half empty: agitation but not Varroa treatment causes cognitive bias in honey bees. *Animal Cognition* 20(2)**,** 233-241. doi: 10.1007/s10071-016-1042-x.

Scollo, A., Gottardo, F., Contiero, B., and Edwards, S.A. (2014). Does stocking density modify affective state in pigs asassessed by cognitive bias, behavioural and physiologicalparameters? *Applied Animal Behaviour Science* 153**,** 26-35. doi: 10.1016/j.applanim.2014.01.006.

Seehuus, B., Mendl, M., Keeling, L.J., and Blokhuis, H. (2013). Disrupting motivational sequences in chicks: Are there affective consequences? *Applied Animal Behaviour Science* 148**,** 85-92. doi: 10.1016/j.applanim.2013.07.008.

Stracke, J., Otten, W., Tuchscherer, A., Puppe, B., and Dupjan, S. (2017). Serotonin depletion induces pessimistic-like behavior in a cognitive bias paradigm in pigs. *Physiology & Behavior* 174**,** 18-26. doi: 10.1016/j.physbeh.2017.02.036.

Titulaer, M., Blackwell, E.J., Mendl, M., and Casey, R.A. (2013). Cross sectional study comparing behavioural, cognitive and physiological indicators of welfare between short and long term kennelled domestic dogs. *Applied Animal Behaviour Science* 147**,** 149-158. doi: 10.1016/j.applanim.2013.05.001.

Verbeek, E., Ferguson, D., and Lee, C. (2014a). Are hungry sheep more pessimistic? The effects of food restriction on cognitive bias and the involvement of ghrelin in its regulation. *Physioly & Behavior* 123**,** 67-75. doi: 10.1016/j.physbeh.2013.09.017.

Verbeek, E., Ferguson, D., Quinquet de Monjour, P., and Lee, C. (2014b). Generating positive affective states in sheep: The influence of food rewards and opioid administration. *Applied Animal Behaviour Science* 154**,** 39-47. doi: 10.1016/j.anbehav.2008.02.014.

Vögeli, S., Lutz, J., Wolf, M., Wechsler, B., and Gygax, L. (2014). Valence of physical stimuli, not housing conditions, affects behaviour and frontal cortical brain activity in sheep. *Behavioural Brain Research* 267**,** 144-155. doi: 10.1016/j.bbr.2014.03.036.

Walker, J.K., Waran, N.K., and Phillips, C.J.C. (2014). The effect of conspecific removal on the behaviour andphysiology of pair-housed shelter dogs. *Applied Animal Behaviour Science* 156**,** 46-56. doi: 10.1016/j.applanim.2014.06.010.

Wheeler, R.R., Swan, M.P., and Hickman, D.L. (2015). Effect of multilevel laboratory rat caging system on the well-being of the singly-housed Sprague Dawley rat. *Laboratory Animals* 49(1)**,** 10-19. doi: 10.1177/0023677214547404.

Wichman, A., Keeling, L.J., and Forkman, B. (2012). Cognitive bias and anticipatory behaviour of laying hens housed in basic and enriched pens. *Applied Animal Behaviour Science* 140**,** 62-69. doi: 10.1016/j.applanim.2012.05.006.
